# Programmable Ultrasonic Sensing System for Targeted Spraying in Orchards

**DOI:** 10.3390/s121115500

**Published:** 2012-11-09

**Authors:** Denis Stajnko, Peter Berk, Mario Lešnik, Viktor Jejčič, Miran Lakota, Andrej Štrancar, Marko Hočevar, Jurij Rakun

**Affiliations:** 1 Faculty of Agriculture and Life Sciences, University of Maribor, 2000 Maribor, Slovenia; E-Mails: peter.berk@uni-mb.si (P.B.); mario.lesnik@uni-mb.si (M.L.); miran.lakota@uni-mb.si (M.L.); jurij.rakun@uni-mb.si (J.R.); 2 Agricultural Institute of Slovenia, 1000 Ljubljana, Slovenia; E-Mail: Viktor.Jejcic@kis.si; 3 Faculty of Computer and Information Science, University of Ljubljana, 1000 Ljubljana, Slovenia; E-Mail: andrej.strancar@fri.uni-lj.si; 4 Faculty of Mechanical Engineering, University of Ljubljana, 1000 Ljubljana, Slovenia; E-Mail: marko.hocevar@fs.uni-lj.si

**Keywords:** air-assisted sprayer, ultrasound, algorithm, programmable microcontroller, spray distribution, orchard

## Abstract

This research demonstrates the basic elements of a prototype automated orchard sprayer which delivers pesticide spray selectively with respect to the characteristics of the targets. The density of an apple tree canopy was detected by PROWAVE 400EP250 ultrasound sensors controlled by a Cypress PSOC CY8C29466 microcontroller. The ultrasound signal was processed with an embedded computer built around a LPC1343 microcontroller and fed in real time to electro-magnetic valves which open/close spraying nozzles in relation to the canopy structure. The analysis focuses on the detection of appropriate thresholds on 15 cm ultrasound bands, which correspond to maximal response to tree density, and this was selected for accurate spraying guidance. Evaluation of the system was performed in an apple orchard by detecting deposits of tartrazine dye (TD) on apple leaves. The employment of programmable microcontrollers and electro-magnetic valves decreased the amount of spray delivered by up to 48.15%. In contrast, the reduction of TD was only up to 37.7% at some positions within the tree crown and 65.1% in the gaps between trees. For all these reasons, this concept of precise orchard spraying can contribute to a reduction of costs and environmental pollution, while obtaining similar or even better leaf deposits.

## 1. Introduction

Nowadays, apple fruit orchards are sprayed mainly with axial fan “mistblower” orchard sprayers owing to their effective axial fan, which offers efficient exploitation under a wide range of variable orchard conditions and training systems. These sprayers are simple, robust, reliable and comparatively low cost in terms of purchase and operation. However, the spray plume generated by axial fan orchard sprayers is prone to spray drift; thus large losses to the atmosphere and ground can occur [1]. The potential for adapting the characteristics of the air stream generated by an axial fan sprayer to different tree canopies is limited. The capacity related to these sprayer features can be overcome by the use of systems for adjusting the applied dose of plant protection products according to the orchard structure (tree row volume—TRV dosing concept), such systems are based on characterization of tree structures by support of sensors, real time signal processing technologies and real time triggering of nozzles. However, the different shapes and sizes of tree canopies, even among the same variety in the orchard, require continual calculation of TRV and adjustment of the applied dose of pesticide to optimize the spray application efficiency [2,3].

For those reasons, in the last 15 years measurement of crop structures has been simplified by the development of a range of non-invasive optical and ultrasonic sampling techniques. In particular, the development of a compact, tractor-mounted light and range detection system (LIDAR) has made it possible to take quick, detailed readings of crop structure [4]. These are suitable for computer processing to calculate a wide range of summary parameters based on a probabilistic interpretation of light transmission and crop interception characteristics [5]. Such a system employs a pulse time-of-flight ranging method, with separate apertures (side-by-side) for an infrared laser diode transmitter and a matched diode light receiver.

The use of ultrasonic devices to measure crop dimensions is not a new idea. Ultrasonic sensors were originally designed to measure distances in industrial environments, where objects are rigid, and the reflection surface is perpendicular to the direction of the ultrasonic wave; therefore, some authors question their usefulness in orchards [6]. Despite these shortcomings, ultrasound sensors are currently being used for the characterization of plant mass and provide good results in certain scenarios. The main advantages of ultrasonic sensors are their robustness and low price. Contrary to the expensive radar system, Gil *et al.* [7] suggested the use of ultrasonic sensors and proportional electro-valves with the corresponding software and automation, which allowed real time modification of the sprayed flow rate adapted to the crop structure of the vineyard.

McConnell *et al.* [8] estimated canopy volume by using several ultrasonic sensors mounted on a vertical mast or on a sprayer driven by a tractor, but the application technologies did not allow this information to be used in real time. Gil *et al.* [7] evaluated a modified orchard air-blast sprayer equipped with three ultrasonic transducers and concluded that savings in pesticide application when using the electronic control system were strongly related to target crop architecture. The same authors found that sprayer control based on target measurement resulted in substantial increases in savings on applied spray liquid.

Moltó *et al.* [9] also applied three ultrasonic sensors for the detection and ranging of geometric information from citrus fruit tree canopies; this enables the application of pesticides in fruit orchards by three different flow rates according to a canopy width estimation made with an ultrasonic sensor. In response to changes in the shape and size of the vines during the growing season Gil *et al.* [7] reported a reduction in spray volume and use of pesticides by up to 57%, while coverage and penetration rates were similar to those from conventional spraying methods. Llorens *et al.* [10] achieved a 58% saving in application volume with the variable rate method, obtaining similar or even better leaf deposits in comparison to the control with an air-blast orchard sprayer. Tumbo *et al.* [11] used ultrasonic sensors to estimate the most relevant geometrical parameters of trees and tree crops *i.e.*, height, width, volume and leaf area and compared these with manual measurements. In [12] the variability in distance estimations in an apple orchard proved to cause interference by sensors whenever these were mounted too close each other; thus it was suggested that sensors be separated more than 60 cm apart in order to avoid high interference effects. In [13] the effect of foliage density and tractor speed on ultrasound measurements was investigated. The software developed to create maps of volume in real time showed the influence of row spacing and age on the accuracy of tree volume measurements.

To now, some of the deficiencies of standard ultrasonic sensors have been overcome by modern signal processing algorithms. Jeon *et al.* [14] decreased errors in sensor laboratory measurements while detecting targets on artificial plants positioned 81.9 cm away by increasing the travelling speeds from 0.8 to 2.0 m·s^−1^.

The objectives of our research were threefold: (a) to analyze the ability of programmable ultrasonic sensors to determine apple tree structure; (b) to investigate the spray volume savings achieved through the use of a target adopted sprayer control system, and (c) to evaluate the efficiency (relative spray deposit) of the proposed spraying system, in comparison to conventional spray application without employment of a TRV based control system.

## 2. Hardware and Software Design

The sensing system consisted of a programmable ultrasonic transceiver and an embedded computer. Experimental work was performed under laboratory and orchard conditions. Sensing tests and the algorithm concepts were set up in the laboratory, and then verified in the orchard.

First, the parameters of the programmable ultrasonic sensing system, such as reflected ultrasonic values, were derived in the orchard. Then, by using these parameters as thresholds, a spraying experiment was performed. For the spraying experiment, a modified axial sprayer was used, while operation was evaluated by deposit measurement by a composite leaf sample method. In the following subsections, design, algorithms of operation and experimental procedure will be discussed in detail.

**Figure 1.**
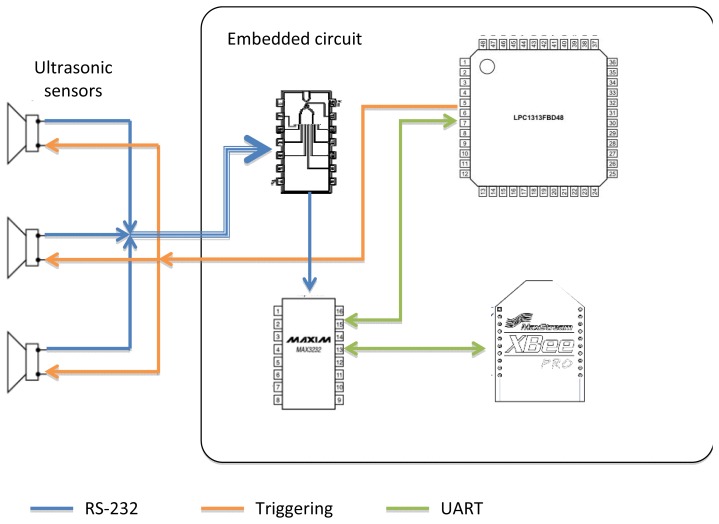
Connection of the major components of the embedded circuit with the ultrasonic sensors.

Figure 1 introduces the concept of interconnection between different parts of the system. On the left side of Figure 1, there are three ultrasonic sensors that are triggered (orange line) by the LPC1343 microcontroller. The sensors then transmit readings by using a RS-232 connection (blue lines), coupled by the AND gate to a MAX232 lever shifter, which is connected to the UART (green line) of the microcontroller. The MAX232 is also connected to an Xbee Pro device that transmits or receives the data from the workstation wirelessly.

### 2.1. Sensor

Sensors were custom made, built around a Cypress PSOC CY8C29466 microcontroller and connected to an ultrasonic PROWAVE 400EP250 transceiver. The PSOC CY8C29466 microcontroller was selected because of its FPGA circuit coupled with a M2C microcontroller. An FPGA circuit enabled us to build a selective amplifier with a 40 dB gain in the frequency range of 40 kHz, needed to amplify the received echoes. Each of the three sensors was connected to the embedded computer and provided cumulative responses over a predetermined range of 1.05 m and a maximum response interval of 0.15 m inside a 1.05 m range. Since there were multiple sensors in the sensing system, we wanted to keep the sensor price as low as possible. Therefore, we used a Cypress PSoC CY8C29466 microcontroller, which also has a highly configurable analog section besides the standard 8-bit microcontroller section. In our case this feature enabled us to build an intelligent sensor with serial output using only one chip–the PSoC, an ultrasonic transducer and a few other components.

The analog section of the PSoC was used for amplifying and band-pass filtering the reflected ultrasonic signal. An envelope of the reflected signal was sampled with an 8-bit AD converter. Other functions such as transmitting ultrasonic bursts, summing the samples and transmitting results over a serial line are realized within the standard microcontroller part. Realizing both the analog and digital part of the sensor with one chip required some tradeoffs. For example, a higher clock frequency also produces more noise in the analog section. In our case we selected a 6 MHz clock, which enabled us to build a 40 dB gain amplifier for the reflected signal. Such gain was suitable for sampling the envelope of the reflected signal with an 8-bit AD converter. The sampling frequency was 15,625 Hz and is limited by the 6 MHz clock frequency (samples were read from AD converter and stored in memory).

### 2.2. Transceiver

**Figure 2.**
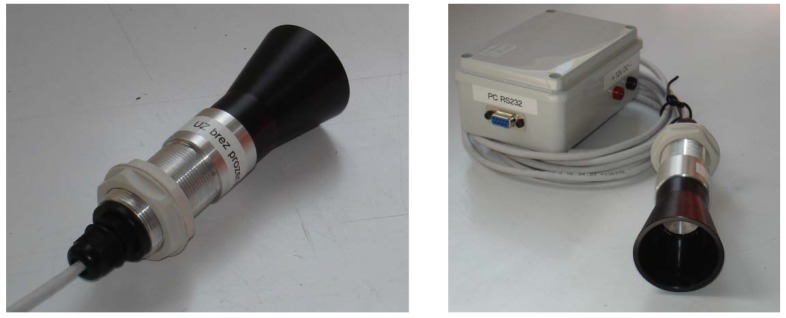
Ultrasonic sensor with ultrasonic transceiver built in the horn. The enclosure includes sensor electronics.

For the ultrasonic transceivers we have chosen PROWAVE 400EP250 transceivers, since these are waterproof and generate a high sound pressure level. They were used to transmit ultrasonic bursts and receive echoes that bounce off nearby objects. The transceivers were connected to the microcontroller, which triggered and analyzed the ultrasonic signals. Each transceiver was equipped with a horn, as depicted in Figure 2. The horns were manufactured from black plastic. Horn length is 45 mm and horn angle, 25°. Ultrasonic transceivers were inserted into the horn, so that they were attached from behind with a permanently elastic silicone kit. The enclosure, to which the horn was attached was manufactured from aluminum, and housed sensor electronics in a watertight compartment. The sensor enclosure was mounted on the sprayer using two nuts.

### 2.3. Embedded Computer

The embedded computer was based on an LPC1343 ARM Cortex-M3 microcontroller, for which a dedicated printed circuit board (PCB) was constructed. The LPC1343 is a low cost and low power microcontroller running at 72 MHz with 8kB SRAM and 32 kB Flash memory, a number of GPIOs and peripherals that include USB 2.0, SSP, I2C, UART and four timers. The PCB included two power regulators, one for 5 V and the other for 3.3 V, three MOSFET transistors that controlled the electro-magnetic valves and a few other basic electronic components.

In addition, support for an Xbee module was included on the PCB. The Xbee modules were used at the development stage to wirelessly send the captured measurements to our workstation, where they were analyzed in order to set the right thresholds on the embedded computer. Furthermore, the Xbee modules were used in simulation test runs, where their role was reversed, and they provided the embedded computer the prerecorded measurements. From the whole Xbee family, we have chosen an XBee Pro series 2 module that has a 50 mW power output, which reaches speeds of up to 250 kbps and has coverage of up to 1.6 km. On one side we connected the module to the RS-232 lines of the LPC1343 microcontroller and on the other to a USB-RS232 converter [15]. We connected the converter to the workstation, where we developed a testing/measuring program using the Matlab [16] programming environment.

### 2.4. Sensing Algorithm

**Figure 3.**
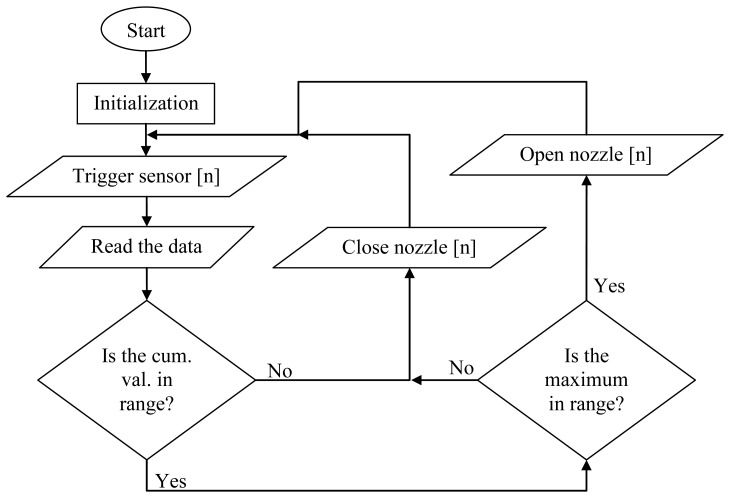
Simplified flow chart (without a delayed circular queue) of an algorithm running on the LPC1343 microcontroller.

Figure 3 represents the “big picture” of how the system works and the role of the LPC1343 microcontroller (without a delayed circular queue). After initialization and triggering of sensors, the system read the data and verified the cumulative response; if it was high enough, we proceeded with further analysis; otherwise, we continued with the next measurement. In the next phase we verified the interval maximum; if the interval maximum was too high and included majority of the response, it measured other targets such as the support column. In a case where the cumulative value was low and the maximum value was below the threshold, we opened the nozzle at the height of the current sensor.

### 2.5. Sensing System

In order to control the operation of the sprayer in automated mode (AM), the sprayer sensing system was tasked to determine the presence or the absence of a target. The sensing system included three ultrasonic sensors with programmable ultrasonic transceivers and the embedded computer described earlier. The sensing data was evaluated, and output was sent to three electro-magnetic valves.

**Figure 4.**
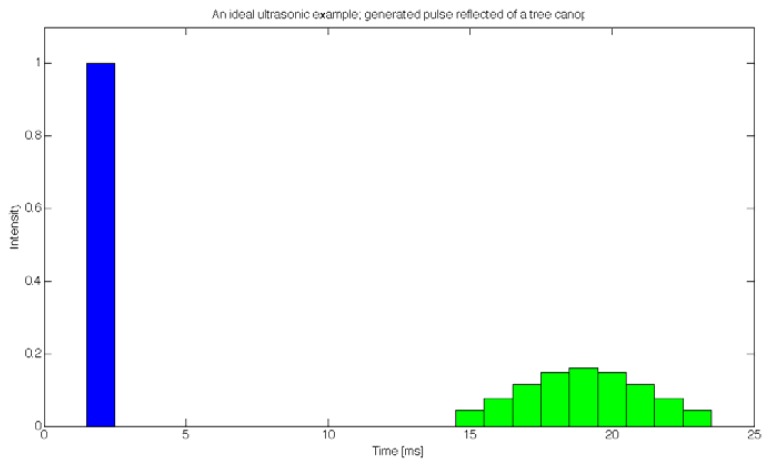
An ideal example depicting a generated ultrasonic burst (blue column) and its reflected version in time series (green columns), where all readings are summed to produce a cumulative response to the canopy measurement.

Each of the sensors sequentially generated a signal that was transmitted to a transceiver in order to produce an ultrasonic sound burst. The transceiver then waited to detect a reflected ultrasonic burst that contained information about canopy structure in a time series evolution of reflected intensity, as depicted by an ideal case in Figure 4; the blue column on the left represents the generated ultrasonic signal with a (normalized) intensity of 1, while the green columns on the right represent the time series evolution, describing the intensities of reflected ultrasound at different distances from the transceiver, measured with nine subsequent samples. One should note that this is an ideal case with time spanning from 0 to 25 ms, which means the canopy would be detected at distances from 2.2 m to 3.6 m. In reality, the range was set from 0.45 m to 1.6 m, which produced nine samples, each spanning 0.15 m in depth, and each corresponding to the thickness of the canopy at a given depth.

**Figure 5.**
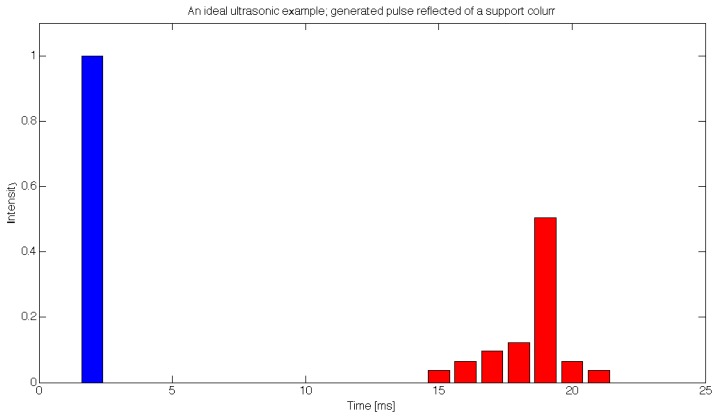
An ideal example depicting a generated ultrasonic burst (blue column) and its reflected version in time series (red columns), where the maximum reading was used to produce a maximum response of the support column measurement.

The first measurements described in the previous paragraph were used to detect a tree canopy. In order to avoid false positive detections caused by targets such as support columns, an additional verification step was included, where extreme reflections were sought, such as the ones in Figure 5; once again, a blue column represents the a generated ultrasonic signal and the red columns represent the reflected ultrasound. The highest red column at 19 ms reveals an obstacle around 5 m from the transceiver with a large surface parallel to the transceiver that produces a good ultrasonic reflection. Since only part of the leaf surface is actually perpendicular to the transceiver, our empirical testing showed that even the thickest canopy did not produce such high reflections. So this can serve as an accurate way to eliminate unwanted objects such as support columns.

Each measurement was summarized by two values and sent to the embedded computer. The first value represented the cumulative value produced by a summation of all nine samples, revealing a possible tree canopy. In order to eliminate false positives, the second value was sent, corresponding to the maximum measurement and revealing extreme local reflections.

In order to prevent ultrasonic sensors from interfering with each other, the embedded computer was used to trigger each of the three transceivers by setting three separate dedicated GPIO lines from logical 0 to logical 1 for a period of 2 ms. This triggered each of the transceivers to capture ultrasonic measurements and sent a replay over a RS-232 line. The same transceivers were used for sending and receiving ultrasonic measurements and were triggered in a subsequent manner, which prevented unwanted false detections that could arise from the signal being detected on a particular transceiver immediately after another transceiver had produced a sound burst.

Simultaneously, the embedded computer calculated the canopy density while processing the response interval. If the calculations were in conformance with normal parameters, they triggered electro-magnetic valves that opened the nozzles. Since the nozzles and the transceivers were not positioned side by side, the embedded computer needed an additional (delayed) circular queue for each nozzle. The queue was filled with values of 1 or 0 that represented the opening/closing of a nozzle, and was read according to the distance between the sensors and the nozzles as well as according to the travelling speed of the sprayer.

These ultrasonic sensors offered the possibility of reprogramming the firmware, whereby each sensor was unique and had to be calibrated. Otherwise, we could end up with sensors that produce different responses from the same target. To prevent this from happening, we used calibration, which involved three measurements:

- when no target is present,
- when a target is just in front of the sensing area and
- when the target is at the end of the sensing area. The calibration parameters as well as ID numbers and the content of the protocol are uploaded to each of the sensors using RS-232 lines prior to its application.

## 3. Experimental Section

### 3.1. General Experiment Information

The basic development and testing of the target measurement and sprayer control system used in this research have been previously described and discussed [17] and will only be briefly outlined in this article.

The spray distribution over sample trees and deposit measurements are the result of experiments carried out in the research orchard of Brdo pri Lukovici (46°10′N, 14°40′E), owned by the Agricultural Institute of Slovenia. Spraying without using ultrasonic control (control spraying mode, CM) was compared to spraying with the ultrasonic control method. This was performed using a prototype ultrasound sprayer control (algorithm) with two different automated spraying modes (AM1 and AM2) according to the statistical analysis of responses, which will be explained precisely in Table 1.

**Figure 6.**
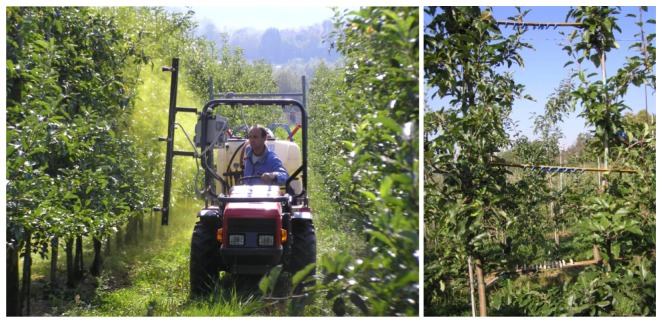
A prototype sprayer during the experiment in the orchard (**left**), detail with measuring positions on the tree (**right**).

The experiments were performed on spindle trained 6-year old “Gala” apple trees, shown in Figure 6, which were grafted onto M9 rootstock and planted at 3.0 × 0.7 m tree spacing. The average height of the trees was 2.5 m. Continuous one side spraying of trees along the tree row was performed from both sides of the row. Five trees and three inter-tree spaces were selected for analysis of spray deposit from trees in the sprayed row.

**Figure 7.**
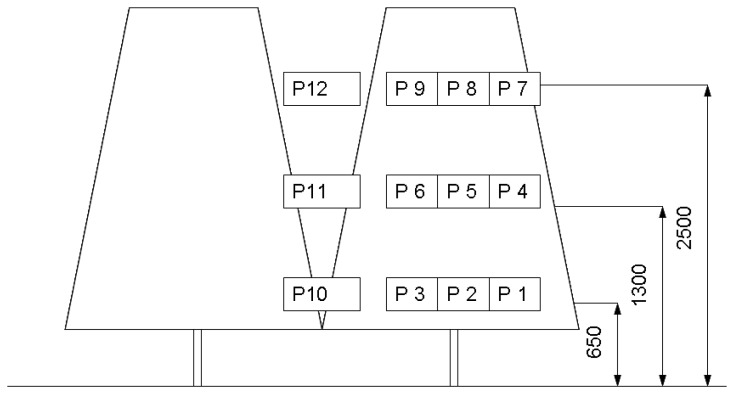
Sampling positions on the tree and between trees.

Each tree (Figure 6(right)) represented one statistical repetition of experimental measurements, with nine positions (P1-P9) analyzed in the canopy and three positions (P10-P12) between trees. The positions (Figure 7) on the tree were selected according to:

- depth: at a distance of 10 cm from the exterior (P1, P4, P7), in the middle at 30 cm from the centre of the trunk (P2, P5, P8), and behind the tree trunk (P3, P6, P9);
- position on the tree: P1, P2, P3 were placed in the bottom part of the tree height (650 mm), P4, P5, P6 in the middle of the tree (1,300 mm) and P7, P8, P9 in the upper part of the tree height (25,00 mm).

An additional three positions (P10-P12) were chosen for measuring deposits in areas between trees according to the height; bottom P10 (650 mm upper the ground), middle P11 (1,300 mm above the ground) and the upper P12 (2,500 mm above the ground).

The experiment was arranged in a single row, from which a 29.35 m long track was selected to ensure constant guiding and meteorological conditions. Any passing to other tree rows would immediately have caused additional variability.

During tests the following values for the meteorological conditions were recorded: temperature 21.9–23.2 °C, relative humidity 45.8%–52.8%, wind speed 1.1–1.7 m·s^−1^ and wind direction 15–45 deg deviation from the perpendicular direction of the sprayer track.

### 3.2. Sprayer

**Figure 8.**
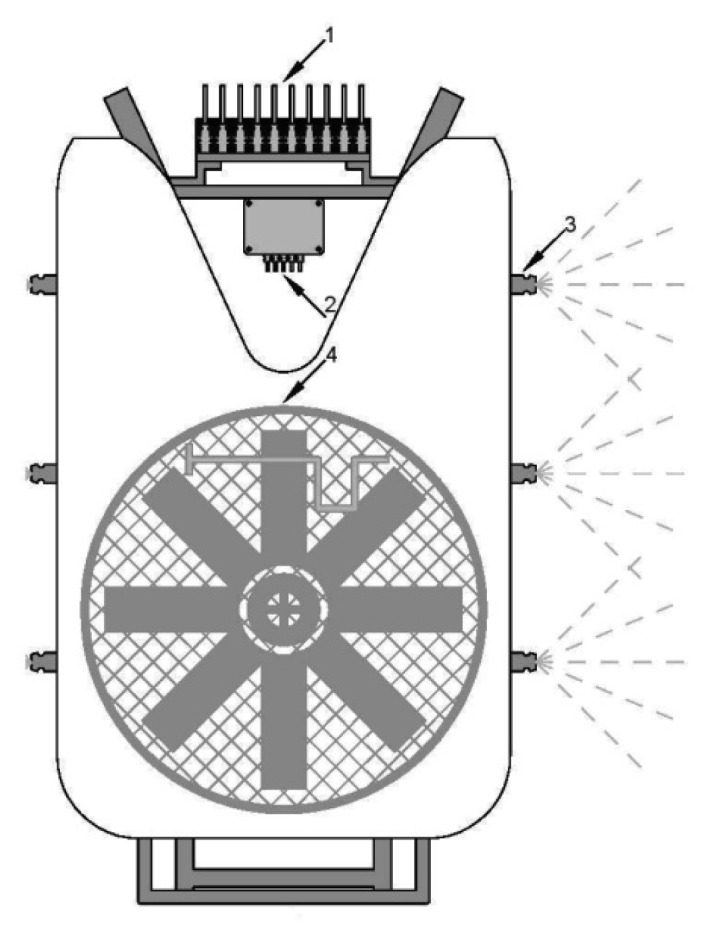
A prototype mounted air-assisted sprayer: (1) electro-magnetic valves, (2) electric box, (3) operating nozzle- left nozzles closed during the experiment, (4) axial fan.

The prototype sprayer was developed by modification-upgrading of a mounted AGP 200 air-assisted sprayer (Agromehanika Kranj, Kranj, Slovenia), equipped with a piston pump and a 200 L tank, a pressure-limiting valve, a blower unit with an axial fan and a nozzle boom around the air outlet (Figure 8). The prototype was fully operational on one side. There were three nozzle sections, with one electric valve mounted in each. Three ultrasonic sensors were placed 280 cm in front of the nozzle plane in the direction of travel (Figure 7). The first bottom nozzle was set at a height of 90 cm, the middle one at 150 cm and the upper one at 210 cm. Each nozzle sprays within an angle 80–90°; therefore, it covers a height of 1 m of the tree crown, if each nozzle is orientated perpendicularly to the tree green wall, and the nozzles are positioned around 0.5 m from the edge of the tree crown. In our case with three nozzles, this was enough to cover a 2.5 m high tree crown, assuming that the lowest 40 cm zone was not sprayed and the neighboring sprays overlap slightly.

To avoid spraying at too low a pressure, an anti-drip device was mounted on each nozzle with an internal spring set to open at 1.5 bar. These devices also enabled the shortening of response times by keeping the pipes full, ready to spray when the pressure exceeded that set with the springs.

Each sensor controlled a single electro-magnetic valve. At the same time a bypass valve in the sprayer manifold allowed the prototype to work also as a conventional sprayer, which was used as a reference in the field tests. All three nozzles of the sprayer were opened in the CM all the time, and none of the three sprayer sections was controlled by the control system, as is the case with the standard radial sprayers already in use. On the other hand, during the AM1 and AM2, the opening or closing of nozzles was controlled online.

### 3.3. Operational Conditions of the Orchard Sprayer

Characterization of the air stream was measured on a stationary sprayer. We used a vane anemometer Schiltknecht MiniAir20 with a 22 mm vane. To ensure proper sampling, air velocities were measured in an axial horizontal direction 500 mm apart from the outlets and the nozzles, where the air jet was wider than the diameter of the anemometer sample volume. Velocity was measured in 20 positions. For all tests, the PTO rotational speed was 540 min^−1^. This gave a mean air volumetric flow rate of 2.80 m^3^·s^−1^ and a mean air velocity of 10.5 m·s^−1^ at a distance of 50 cm from the sprayer.

The sprayer was equipped with three hollow cone nozzles (Lechler TR yellow) operating with a pressure drop of 10.0 bar, to give total spray flow rates of 4.35 L·min^−1^. Thus, the maximum range of values for the applied spray volume per unit of ground area was 226 L·ha^−1^, when all three nozzles were opened. The sprayer settings (Table 2) were the same for all operating modes.

The pump used was a four piston semi-hydraulic diaphragm pump model (BM 65/30, Agromehanika, Kranj, Slovenia) with a volume flow of 60 L·min^−1^ at a selected rotational speed of 540 min^−1^.

### 3.4. Sprayer Flow Rate Calculation

The different components and the control system were tested and fine-tuned in the laboratory and on artificial targets; later, when the sprayer prototype was fully assembled, it was tested in the orchard. This process resulted in a real-time system for the continuous opening and closing of all three nozzles according to the tree canopy structure. The real-time flow rate of each electro-valve controlled nozzle of the sprayer prototype was computed as follows:

(1) $$q=p\cdot (\frac{a\cdot v\cdot V_{r}}{600\cdot N})$$

where *a* is a working width, here we considered only one side of the sprayer to be operational, and working width was reduced by half; *v* is the speed in km·h^−1^; *V_r_* is the volume application rate for the orchard in L·ha^−1^; *N* is the number of nozzles; and *p* is the reduction coefficient of the maximum flow rate given by the following equation:

(2) $$p=\frac{\sum t_{i}}{\sum t}$$

where Σ*t_i_* is the sum of actual opening time for each nozzle, Σ*t* is the sum of maximum possible opening time for all nozzles.

### 3.5. Measurement of Deposits

Deposit and spatial distribution of spray liquid were measured using tartrazine (Citronin yellow, ETOL, Celje, Slovenia) as a spray tracer [18,19] because it has a high recovery rate and high photostability [20]. Deposits and spatial distribution of spray savings were measured on composite leaf samples at a rate ranging from 0.15 to 3.90 μg·cm^−2^, depending on the treatment (Table 3) by following the protocol established by [20]. Samples were taken from the 12 positions of selected trees in five replications, so that each sample contained five leaves. Every new series of leaves for deposit measurements was fastened by clothespins, which were fixed in exactly the same positions throughout all three sprayer operating modes. This ensured exactly the same positions of leaves for both modes of spraying and reduced the variability caused by leaf position at the points of deposit analysis. After each experiment, the leaves were collected from the clothespins and placed in plastic bags, taken to the laboratory and stored in a dark, cool place before processing. Prior to the application, 100 leaves were picked from each individual block as blank samples, in order to determine the leaf area index (LAI), average weight and possible presence of tartrazine. The concentration of the tracer in the applied spray was 5 g·L^−1^ in all three treatments. In all cases, values of tracer concentration in the blank samples were less than the detection limit of the spectrophotometer (<0.01 ppm).

After drying of the deposit, the leaves were removed from the clothespins and put into plastic bags. Once collected, all plastic bags were placed in a dark container and stored in a refrigerator until the extraction process. Leaf samples were washed with 100 mL distilled water, shaken in the same plastic bag as collected, and three samples of 2 mL were taken for determining the concentration of tartrazine on a Varian CARY 50 BIO spectrophotometer. We followed the experimental procedures presented by [20]. Nevertheless, previous tests were made in the laboratory to confirm the accuracy of the methodology, especially in relation to tracer recovery from apple leaf samples, whereby the theoretical and normalized deposit was calculated according to the procedure described by [19]. The tartrazine recovery rate was determined to be 90%.

All data was transferred from the Varian spectrophotometer into formatted computer spread-sheets (Microsoft Excel) before statistical analysis of variance (3 treatments × 5 repetitions × 12 locations) was performed, using the Statgraphics Centurion XVI Package Program (StatPoint Technologies, Inc. Warrenton, VA, USA).

The theoretical deposit per unit leaf area (*Td*) in (μg·cm^−2^) was calculated by dividing the application rate (*Q*) in (L·ha^−1^) and the tracer concentration (*T_c_*) in (g·L^−1^) by the total leaf area index (*LAI*) in (m^2^·ha^−1^), according to the following equation:

(3) $$Td=\frac{Q\cdot T_{c}}{L\cdot 10^{4}}$$

The amount of spray deposited per unit leaf area in a particular treatment (*Md*) was calculated by dividing the tracer concentration in the washing solution of sample (*T_cl_*) and *w* the amount of deionized water in (mL), by the total leaf area of the sample *L_a_* in (cm^2^), according to the following equation:

(4) $$Md=\frac{T_{cl}\cdot w}{L_{a}}$$

Since the tracer application rates (*T_cl_*) were not the same for all treatments, because nozzles were not opened for the same time in all treatments, a normalized deposit (*Nd*) was then calculated according to Equation (5), by dividing the measured deposit with the theoretical deposit (*Td*) of a particular treatment:

(5) $$Nd=\frac{Md}{Td}$$

The normalized deposit procedure enables comparison between the different modes of spraying and was based on the total amount of tracer applied per TRV. This procedure has been previously applied by [1,21], where comparisons between different sprayers and/or field conditions were arranged.

## 4. Results and Discussion

### 4.1. Calibration of the Sensing System

**Figure 9.**
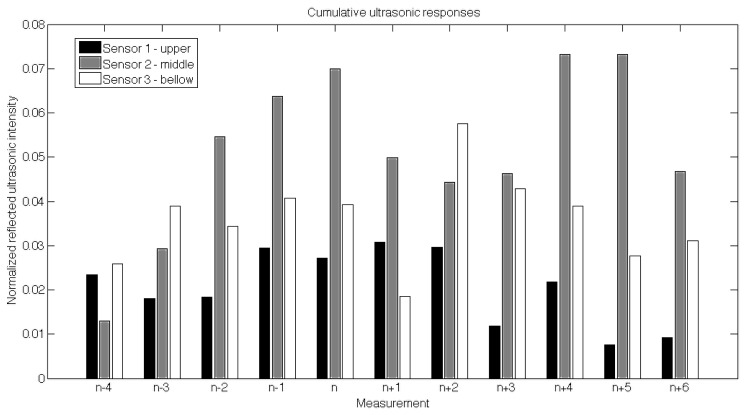
Cumulative ultrasonic responses for each sensor recorded during the calibration run in the orchard around two random tree canopies.

Prior to the field experiment, calibration and fine tuning of all components were adjusted according to the ultrasound test measurements in the same orchard. Two figures (Figures 8 and 9) were derived from the test examples depicting two different test runs with 11 successive measurements that best describe each situation; a successive measurement of a tree canopy and a successive measurement of a tree canopy with a support column. Figure 9 consists of 11 successive cumulative responses for all three sensors, where all measurements are designated as n ± number; an “n − 4” corresponding to the first and an “n + 6” to the last measurement. The measurement “n” corresponds to a situation where the sensors were just in front of the tree canopy. Each measurement was assembled from three columns corresponding to the cumulative (the integral of all responses). As can be seen, while approaching the measurement “n”, the middle sensor value increases. As the sensors move away from the tree, the responses get lower. When we approach the second tree (between “n + 4” and “n + 5”) the same pattern is again detected. Since the tree crown is most dense in the middle part of the tree, the ultrasound responses of the middle sensor showed higher values than the upper and the lower ones. However, besides the cumulative responses, we enhanced the scanning of the tree structure and spraying with an additional measurement. As described earlier, we included maximum responses to separate support columns from the thick, dense tree canopy.

**Figure 10.**
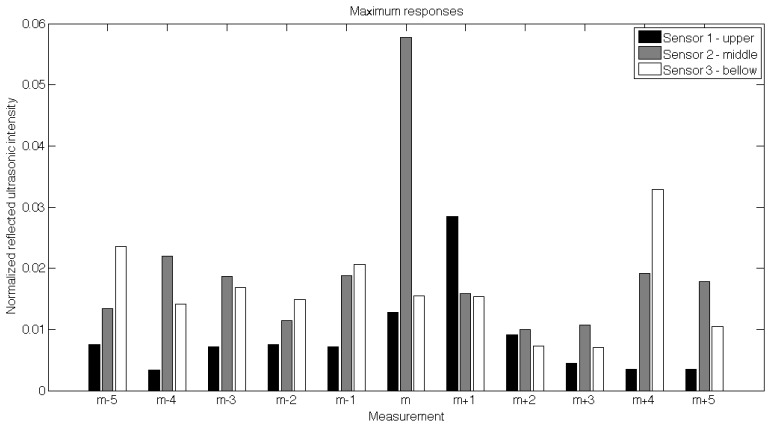
Maximum responses for each sensor recorded during the calibration run in the orchard around one random support column.

Figure 10 shows a sample of 11 successive maximum responses of all three sensors (upper, middle and bottom sensors) during sensing a randomly selected support column (m). Each measurement is assembled from three columns corresponding to maximum responses.

As can be seen, on the “m” measurement the maximum values of the second sensor suddenly doubled, which is a clear evidence of supporting column presence. Since the sensors were triggered in a subsequent manner, the highest (complete) response was recorded by the middle sensor, but partial reflection is also visible on the m + 1 sample from the sensor positioned above. According to the analysis of the calibration runs, we were able to program the LPC1343 microcontroller with the proper threshold values. The thresholds (Table 3) were set for two different automated operation modes (AM1 and AM2) according to the statistical analysis of responses (average and standard deviation) of multiple calibration runs on the row planted with 42 trees.

Thresholds were implemented according to the following reasoning:

- individual calibrations of sensors were performed in order to guarantee the same responses generated by all sensors for the same target,
- maximum response of each sensor was set for both AM1 and AM2, as shown in Table 3, in order to detect the support columns. That approach could proceed because all elements were the same in the calibration runs-cumulative response of each sensor was selected according to previous experiments reported by [7,10], in which accurate spray distribution was achieved at 58% and 57% closing time of nozzles. For this reason, we set the cumulative response according to average values and standard deviations, so the nozzles should be closed 50% of the time (AM1) and 60% of the time in the case of AM2.

As can be seen from in Table 3, the highest cumulative threshold was selected for the middle sensor, followed by the bottom one, which is strongly connected to the tree silhouettes, which are most dense in those two particular zones. On the other hand, the upper sensor was set at a threshold of 0.017 (AM1) and 0.0178 (AM2), since there are already more empty spaces on this part of the tree.

When the cumulative response was higher than the thresholds, the computer proceeded with the final step of the algorithm that verified the presence of supported columns, which corresponds to maximum response. For this reason, the threshold values for all tree sensors were set similarly, at 0.5 (AM1) and 0.6 (AM2).

### 4.2. Reduction of Spray Delivered Per Area Unit

Total savings from the programmable ultrasound sensing system were evaluated as the reduction of spray delivered per area unit for each individual test.

The working time and real-time flow rate of each particular nozzle for a test track are shown in Table 3. The effective working time for each nozzle was calculated as the sum of all opening times from the stored data of the electro-magnetic valves' open/closed status during the driving along the track, while the real-time flow rate was calculated according to the driven path and time. The procedure of turning the nozzles on/off was explained in Section 2.7.

Given the average operating time on the 29.35 m long experimental field track of 58.70 s per nozzle in the CM, 37.49 s within the AM 1 and 30.41 s within the AM 2, the calculated spray savings were, on average, 36.13% for all three nozzles together in AM 1 and 48.19% in AM 2.

A significant reduction of the average real-time flow rate per nozzle according to the selected A/C index from l.45 L·min^−1^ to 0.92 L·min^−1^ (37% reduction) was achieved in AM 1 and 0.75 L·min^−1^ in AM 2 (48% reduction), in comparison with CM. In AM 1 mode the upper sensor was open for 19.72 s, the middle one for 46.96 s and the bottom one for 45.79 s; thus, we assume that tree silhouettes of the spindle formed trees with conical shapes and interspaces were, on average, detected correctly for all three heights at the same time.

In the AM 2 mode the upper and bottom sensors were closed for longer time than in AM1 because higher cumulative response thresholds indeed control the spray flow in a different way, as in the case of AM1 when comparing the middle and bottom part of the trees. It means that on a similar scene the nozzles were closed for a longer time. But in AM2 the open time for the area of the middle sensor was unexpectedly higher than in the bottom part, although the selected trees had a spindle shape with approximately the same width in the middle and bottom part, which should result in similar closing times. Those differences may be viewed as a measurement uncertainty of the system caused by various sources: (1) uneven ground, (2) not driving exactly in the middle of rows, (3) moisture, temperature dependency of the sensor response, (4) the influence of wind and (5) slightly variable driving speed.

We can assume that opening times from our experiment are 15% (AM1) and 27% (AM2) shorter that those reported by [16,21], while at the same time maintaining sufficient spray distribution. Thus, our findings are comparable with the 28% spray savings in a high density pear plantation reported in [9]. On the other hand, in our case it was impossible to reach 68% reduction of pesticide application as reported by [3] for an older olive plantation, or the 57% reduction in the total amount of applied volume according to the instantaneous ultrasound measurements as indicated by [5], since in our experiments the trees were trained in spindle and are more uniform and dense.

### 4.3. Spray Deposition

The quality of spray distribution determined by spectrophotometric measurements of tartrazine deposits on the leaf samples was expressed in μg/cm^2^ as well as in the form of a normalized deposit, whereby in CM the maximum theoretical deposit of 4.63 μg/cm^2^ is assumed as 1 (100%), 2.95 μg/cm^2^ in AM1 and 2.39 μg/cm^2^ in AM2. For estimating the data according to Equations (3) and (4), average leaf area of 28.55 cm^2^, average leaf mass of 0.795 g and LAI 2.4 m^2^·ha^−1^ were used.

The difference in the theoretical starting-points of all three modes is due to the 63% nozzle opening time during AM1 and 52% nozzles opening time during AM2 (Table 3). Table 4 shows a summary of the results obtained in the orchard during tests on apple trees. In CM, on average, there was a higher deposit (1.54 μg/cm^2^—0.33 normalized deposit) in the upper tree positions (P7, P8 in P9) than in the middle (P4, P5 in P6) and bottom positions (P1, P2 in P3), which means that an important loss of spray appeared in this part of the tree, as is common with conventional spraying.

The measured deposit of ‘all positions’ reached 0.54 μg/cm^2^ (0.18 normalized deposit) in AM 1, which is significantly lower than in the case of CM and AM2. However, owing to the 66.4% lower amount of spray flow through the above nozzles, the spray savings reduced the normalized deposit significantly in the upper tree positions, whereby the normalized deposit of the upper leaves was only 0.16. It seemed that the programmable threshold values were set too high, thus turning off nozzles at even a small presence of branches or leaves. On the other hand, in the between-tree positions, the significant reduction of normalized deposit means that the sensing system detected gaps between the trees and closed the nozzles as expected.

In AM 2 the measured deposit of “all positions” was 0.83 μg/cm^2^ (0.35 normalized deposit), which is significantly higher than in the case of AM1, but unchanged to CM. Thus the 48.8% spray savings did not influence the normalized deposit, which remained equal to CM. We believe the more accurate sensor measurements were done in AM2, since selected threshold values proved to detect tree density better than in the case of AM1, where threshold values were lower by approximately 0.0006. We can conclude that our findings are very close to the trials of [22], who obtained no significant differences in tracer deposition between a sensor-based and a conventional application technique for apple scab, pear psylla and leaf bud mite control. However, many trials are still required in order to establish a site-specific adaptive threshold values.

## 5. Conclusions

Detailed analysis of ultrasonic tree canopy sensing by PROWAVE 400EP250/Cypress PSOC CY8C29466 combination-based sensors controlled by a LPC1343 embedded computer was performed to increase the accuracy of target detection in orchards. The use of programmable microcontrollers enables the setting of a combination of threshold values for cumulative response and the integral of maximal response. Based on much fine-tuning, two operational modes AM1 (cumulative response threshold at average value ± σ) and AM2 (cumulative response threshold at average value) were chosen for performance of the field experiments. The programmable ultrasonic electronic control system for proportional spray application AM1 showed in total a 36.1% saving of spray per nozzle and area unit and AM2 48.19% in comparison to CM (no sensor guidance). These savings were achieved without significant reduction of tracer deposit at any tree position except on the upper tree P8 and P9 positions in AM1. In contrast, in the upper mid-tree position P12 in AM2 the deposit was increased, which made the approach interesting for further developments. However, in the reduction of spray deposit in areas between trees, the AM1 (65.1% reduction) provided better results than AM2 (22.7% reduction).

An algorithm with cumulative and maximal response thresholds was used, which proved suitable for fine tuning the ultrasound sensitivity of a low-cost sensor system. However, these values can vary from one orchard to the next and depend on the fruit type, tree age, growing system and even the humidity conditions. For this reason, potential extensions to the system seem appealing and will be implemented and tested in our future work. It will be built around a fuzzy logic control, which has recently been widely introduced in agricultural computers and technology. Based on the readings from the sensors, the system could adopt the liquid pressure in the sprayer and opening of the nozzles, according to the criteria of fuzzy logic. In order to achieve this, we will implement a pulse-width-modulation approach to controlling the electro-magnetic valves, where the pulses are controlled by the density of the tree canopy. Based on the ultrasound responses, the microcontroller would generate wider or narrower pulses, which in effect control the flow rate. This algorithm could prevent excessive flow of drops from the nozzles towards the tree crowns instead of limited flow in all cases where small objects, such as individual branches, are detected.

## Figures and Tables

**Table 1.** Normalized cumulative and maximum responses selected for AM1 and AM2

**Sensor**	**Normalized Cumulative Response**				**Normalized Maximum Response**			

	**Average**	**σ**	**AM1**	**AM2**	**Average**	**σ**	**AM1**	**AM2**
Sensor #1	0.023	±0.015	[0.017, 1]	[0.017, 1]	0.012	±0.011	[0, 0.056]	[0, 0.060]
Sensor #2	0.048	±0.022	[0.043, 1]	[0.043, 1]	0.021	±0.011	[0, 0.052]	[0, 0.056]
Sensor #3	0.040	±0.019	[0.033, 1]	[0.035, 1]	0.018	±0.010	[0, 0.050]	[0, 0.059]

**Table 2.** Operational parameters during treatments.

	**Operational Parameters**
Nozzle serial number	Lechler TR 80-02
Colour	Yellow
No. of active nozzles per side	3
Pressure (bar)	10 bar
Spray flow rate per nozzle (L·min^−1^)	0–1.45
Spray flow rate all nozzles (L·min^−1^)	0–4.35
Forward speed (km·h^−1^)	3.6
Working width (m)	3.00
Reference application rate (L·ha^−1^)	226
PTO speed (rev·min^−1^)	540
Volumetric air flow rate (m^3^·s^−1^)	2.80

**Table 3.** Average working time (s) and real-time flow rate (L·min^−1^) for automated (AM1 and AM2) and control (CM) spray distribution.

**Nozzle/Deflector Position**	**Control (CM)**			**Automated 1(AM 1)**			**Automated 2(AM 2)**		

	**Open** **Time** **(s)**	**Close** **Time** **(s)**	**Real-Time** **Flow Rate** **(L·min^−1^)**	**Open** **Time** **(s)**	**Close** **Time** **(s)**	**Real-Time** **Flow Rate** **(L·min^−1^)**	**Open** **Time** **(s)**	**Close** **Time** **(s)**	**Real-Time** **Flow Rate** **(L·min^−1^)**
1st upper	58.70	0.00	1.45	19.72	38.97	0.34	9.16	49.54	0.23
2nd middle	58.70	0.00	1.45	46.96	11.74	0.80	46.72	11.98	1.15
3rd bottom	58.70	0.00	1.45	45.79	12.91	1.13	35.34	23.36	0.87
Average	58.70	0.00	1.45	37.49	21.21	0.92	30.41	28.29	0.75

Index A/C	58.70	-	-	-	-	0.63	-	-	0.52

**Table 4.** Comparison between the tartrazine tracer deposit (μg/cm^2^) in control and automated (AM) spray distribution.

**Position**	**CM**		**AM1**		**AM2**	

	**Measured** **Deposit** **μg/cm^2^**	**Normalized** **Deposit** *	**Measured** **Deposit** **μg/cm^2^**	**Normalized** **Deposit** **	**Measured** **Deposit** **μg/cm^2^**	**Normalized** **DEPOSIT** ***
Bottom tree P1, P2, P3	0.94 C	0.20 A	0.51 B	0.19 A	0.35 A	0.15 A
Middle tree P4, P5, P6	1.23 C	0.27 A	0.53 A	0.18 A	0.86 B	0.38 AB
Upper tree P7, P8, P9	1.54 C	0.33 B	0.47A	0.16 A	0.82 B	0.31 B
All on the tree	1.24B	0.27 B	0.51A	0.17 A	0.67AB	0.28 B
Between trees P10, P11, P12	1.83 C	0.66 B	0.62A	0.23 A	1.21B	0.51 B

All positions	1.39 C	0.30 B	0.54 A	0.18 A	0.83 B	0.35 B

* 4.63 μg/cm^2^ = 1 = 100% of theoretical CM,

** 2.95 μg/cm^2^ = AM 1,

*** 2.39 μg/cm^2^ = AM 2

Normalized deposit = measured deposit/theoretical deposit *

A, B, C. difference between CM, AM1 and AM2 spraying modes (t-test; α = 0.05)
